# Identification of immune-related genes as prognostic factors in bladder cancer

**DOI:** 10.1038/s41598-020-76688-w

**Published:** 2020-11-12

**Authors:** Jie Zhu, Han Wang, Ting Ma, Yan He, Meng Shen, Wei Song, Jing-Jing Wang, Jian-Ping Shi, Meng-Yao Wu, Chao Liu, Wen-Jie Wang, Yue-Qing Huang

**Affiliations:** 1 Department of Oncology, Changzhou Traditional Chinese Medical Hospital, Changzhou, 213003 Jiangsu People’s Republic of China; 2 Department of Oncology, Jining Tumour Hospital, Jining, People’s Republic of China; 3 Department of Radio-Oncology, The Affiliated Suzhou Hospital of Nanjing Medical University, Suzhou, 215001 Jiangsu People’s Republic of China; 4 Department of Oncology, The First Affiliated Hospital of Soochow University, Suzhou, 215006 Jiangsu People’s Republic of China; 5 Department of Gastrointestinal Surgery II, Renmin Hospital of Wuhan University, Wuhan, People’s Republic of China; 6 Department of Oncology, Taizhou Hospital of Traditional Chinese Medicine, Taizhou, People’s Republic of China; 7 Department of Urology, The Affiliated Suzhou Hospital of Nanjing Medical University, Suzhou, 215001 Jiangsu People’s Republic of China; 8 Department of General Practice, The Affiliated Suzhou Hospital of Nanjing Medical University, Suzhou, People’s Republic of China

**Keywords:** Cancer, Cancer genomics, Tumour biomarkers, Tumour immunology, Urological cancer, Immunogenetics, Immunotherapy, Tumour immunology

## Abstract

Bladder cancer is one of the most common cancers worldwide. The immune response and immune cell infiltration play crucial roles in tumour progression. Immunotherapy has delivered breakthrough achievements in the past decade in bladder cancer. Differentially expressed genes and immune-related genes (DEIRGs) were identified by using the edgeR package. Gene ontology annotation and Kyoto Encyclopedia of Genes and Genomes (KEGG) pathway analyses were performed for functional enrichment analysis of DEIRGs. Survival-associated IRGs were identified by univariate Cox regression analysis. A prognostic model was established by univariate COX regression analysis, and verified by a validation prognostic model based on the GEO database. Patients were divided into high-risk and low-risk groups based on the median risk score value for immune cell infiltration and clinicopathological analyses. A regulatory network of survival-associated IRGs and potential transcription factors was constructed to investigate the potential regulatory mechanisms of survival-associated IRGs. Nomogram and ROC curve to verify the accuracy of the model. Quantitative real-time PCR was performed to validate the expression of relevant key genes in the prognostic model. A total of 259 differentially expressed IRGs were identified in the present study. KEGG pathway analysis of IRGs showed that the “cytokine-cytokine receptor interaction” pathway was the most significantly enriched pathway. Thirteen survival-associated IRGs were selected to establish a prognostic index for bladder cancer. In both TCGA prognostic model and GEO validation model, patients with high riskscore had worse prognosis compared to low riskscore group. A high infiltration level of macrophages was observed in high-risk patients. OGN, ELN, ANXA6, ILK and TGFB3 were identified as hub survival-associated IRGs in the network. EBF1, WWTR1, GATA6, MYH11, and MEF2C were involved in the transcriptional regulation of these survival-associated hub IRGs. The present study identified several survival-associated IRGs of clinical significance and established a prognostic index for bladder cancer outcome evaluation for the first time.

## Introduction

With an estimated 81,190 newly diagnosed cases and 17,240 deaths occurring in 2018, bladder cancer (BC) ranks as the fifth most common cancer in the USA^1^. Multiple risk factors are involved in the occurrence and development of bladder cancer, among which tobacco smoke is a principal risk factor^2^. Previous studies have reported that approximately 70% of BCs are at a non-muscle-invasive stage at diagnosis, and bladder resection is the standard treatment for these patients^3^. For metastatic or unresectable bladder cancer, platinum-based chemotherapy has been certified as the standard first-line treatment^4^. However, traditional chemotherapy has shown no effect on prolonging overall survival (OS)^5^.

The correlation between clinical outcomes and the density of tumour immune infiltration has been confirmed in previous studies^6^. Strong evidence demonstrates that innate immune cells, including lymphocytes, macrophages, neutrophils and dendritic cells, play a crucial role in cancer development^7^. With complex interactions with tumour cells in the tumour microenvironment, these immune cells not only fail to work against tumour cells but also mediate immune tolerance and act as tumour promoters^8^.Immunotherapy has delivered breakthrough achievements in several cancer types in the past decade^9–12^. Outstandingly, recent studies and clinical trials have made encouraging progress in immunotherapy for metastatic bladder cancer since the U.S. The Food and Drug Administration (FDA) approved the application of immune checkpoint agents in bladder cancer treatment. For instance, MPDL3280A (anti-PD-L1) has been demonstrated to show remarkable activity in metastatic bladder cancer^9^.

While immunotherapy has improved the outcome of bladder cancer, the immunogenomic landscape of bladder cancer still remains unknown. In the present study, we aimed to investigate the impact of immune-related genes (IRGs) on prognosis prediction and identify potential prognostic biomarkers for bladder cancer.

## Result

### Identification of differentially expressed genes (DEGs) and differentially expressed IRGs (DEIRGs)

**Figure 1:**
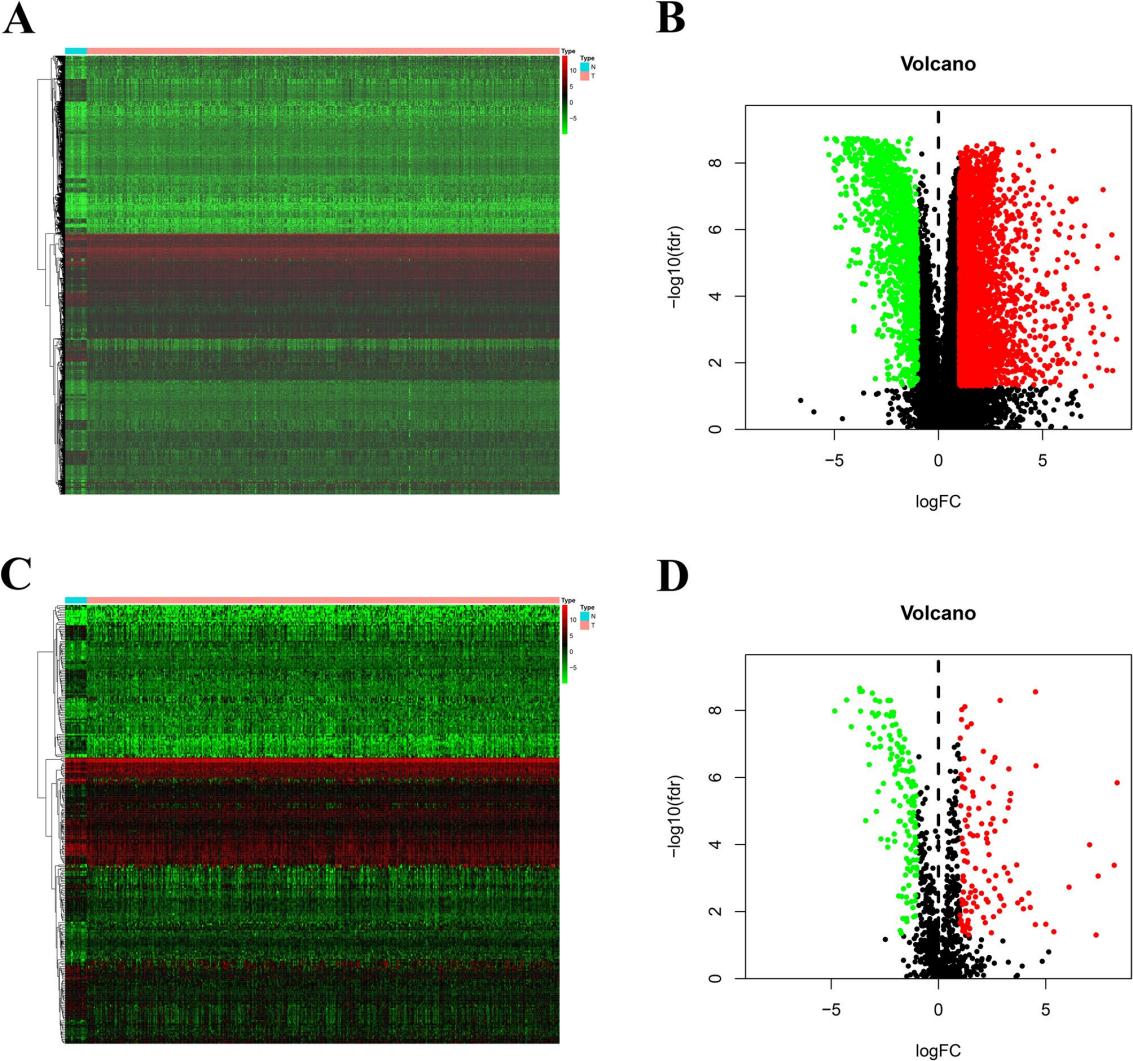
Differentially expressed genes (DEGs) and immune-related genes (DEIRGs): The heatmap (**A**) and volcano (**B**) of DEGs between bladder cancer tissues and no-cancer tissues. The heatmap (**C**) and volcano (**D**) of DEIRGs between bladder cancer tissues and no-cancer tissues.

The edgeR package in the R language was used to further analyse the data. To identify differentially expressed genes, a |log2 fold change (FC)|> 1.5 and a false discovery rate (FDR) adjusted to a *p* < 0.01 were set as the thresholds. Additionally, heat maps and volcano maps of the differentially expressed RNAs were generated by using the gplots and heatmap packages in the R platform. Among the 4880 differentially expressed genes, 3458 up-regulated genes and 1422 down-regulated genes were identified (Fig. 1A,B). Among the set of differentially expressed genes, 259 differentially expressed IRGs (DEIRGs) were screened out, including 140 up-regulated and 119 down-regulated genes (Fig. 1C,D). In Gene Ontology (GO) analysis of DEGs, a total of 10 significantly enriched pathways were obtained (Supplementary material 1). The results indicated that the extracellular region was the most frequent GO biological process category (*p* < 0.05) (Supplementary material 2). In the KEGG pathway analysis, a total of 10 significantly enriched pathways were obtained. Among the top 10 pathways, “MAPK signalling pathway”, “IL-17 signalling pathway”, “cytokine-cytokine interaction receptor”, “neuroactive ligand-receptor interaction” and “viral protein interaction with cytokine and cytokine receptor” were identified as the top 5 enriched pathways. A visual network between IRGs and the top 5 KEGG pathways was constructed by using Cytoscape v3.6.1 (Supplementary material 3). Cytokine-cytokine receptor interaction was the most frequent KEGG pathway category (*p* < 0.05) (Supplementary material 4). In GO analysis of DEIRGs, the extracellular region was the most frequent GO biological process category (*p* < 0.05) (Supplementary material 5). In the KEGG pathway analysis, the MAPK signalling pathway was the most frequent KEGG pathway category (*p* < 0.05) (Supplementary material 6).

### Identification of differentially expressed transcription factors

**Figure 2:**
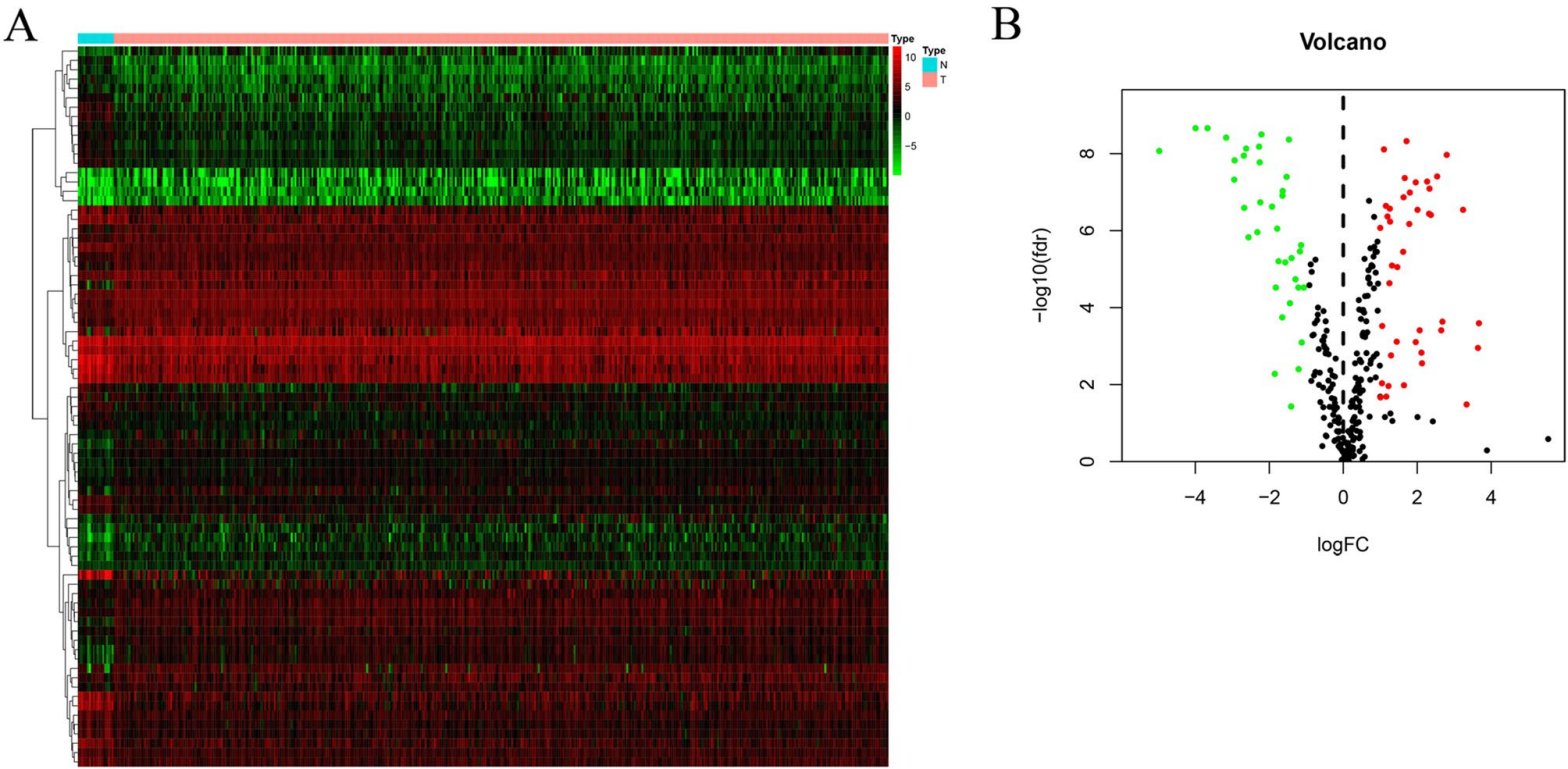
Differentially expressed transcription factors (DETFs): the heatmap (**A**) and volcano (**B**) of DETFs between bladder cancer tissues and no-cancer tissue.

To investigate the regulatory mechanisms of these IRGs, we further examined the expression profiles of 77 transcription factors (TFs). Among the 77 differentially expressed TFs, 34 up-regulated TFs and 41 down-regulated TFs were identified (Fig. 2).

### Evaluation of clinical outcomes

**Figure 3:**
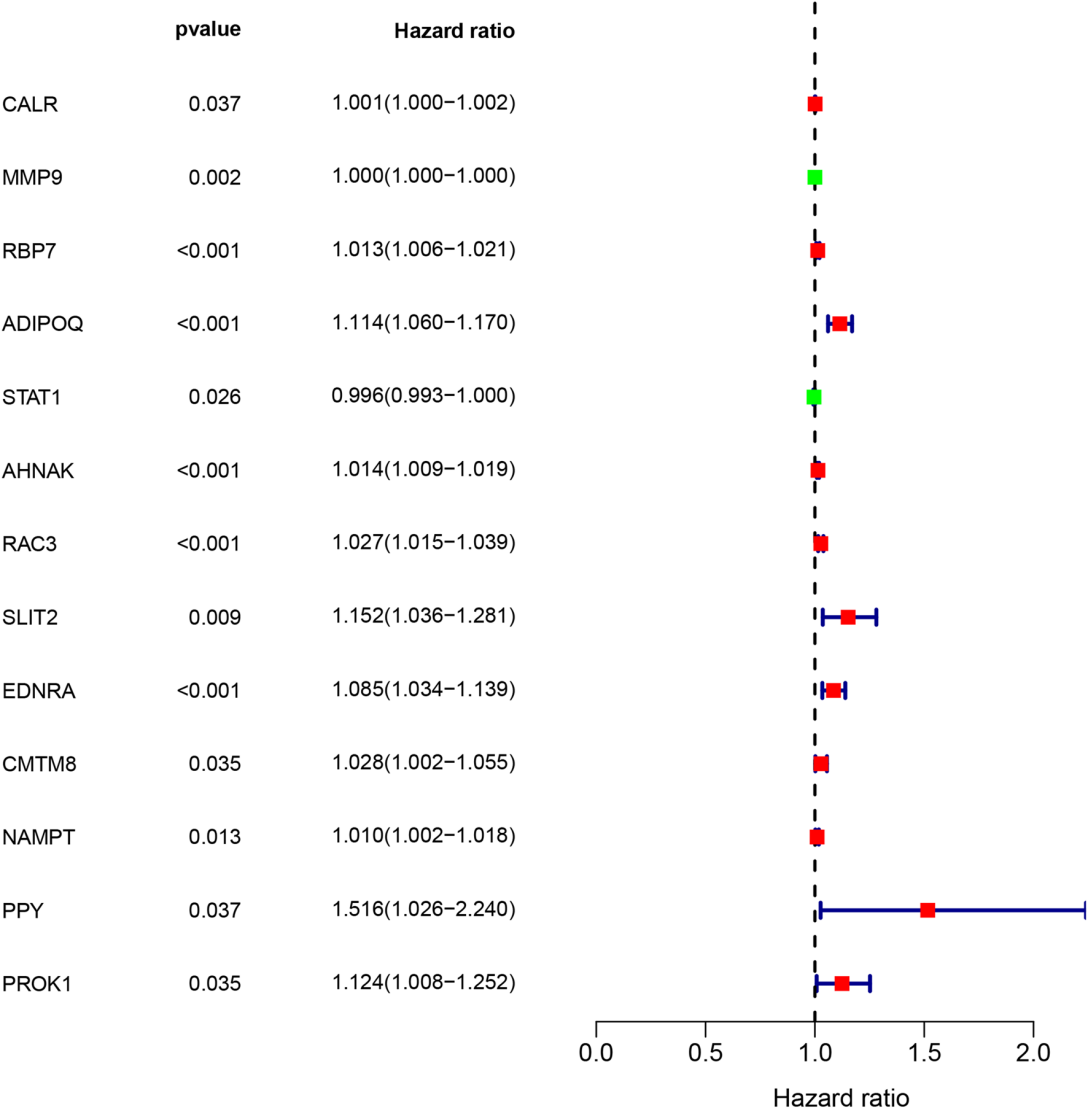
Prognostic values of survival-associated IRGs: the forest plot of survival-associated IRGs.

**Figure 4:**
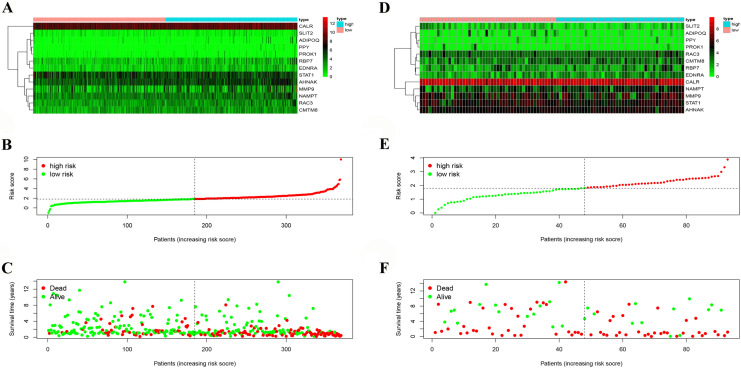
Development of the immune-related gene prognostic index (IRGPI) and validation model: (**A**) Heatmap of expression profiles of included genes in IRGPI. (**B**) Rank of prognostic index and distribution of groups in IRGPI. (**C**) Survival status of patients in different groups in IRGPI. (**D**) Heatmap of expression profiles of included genes in validation model. (**E**) Rank of prognostic index and distribution of groups in validation model. (**F**) Survival status of patients in different groups in validation model.

Based on univariate Cox regression analysis, we identified 27 survival-associated IRGs, constructed a prognostic index containing 13 survival-associated IRGs by multiple Cox regression analysis (Fig. 3), and separated BC patients into two groups (high risk score group and low risk score group) with regard to OS and median risk score (Fig. 4A–C). The prognostic model was verified by a validation prognostic model based on the GEO database. In the validation model (Fig. 4D–F).The survival risk score was calculated as follows:

$$ Survival\;Risk\;Score \left( {SRS} \right) = \mathop \sum \limits_{i = 1}^{k} \left( {C_{i} \times V_{i} } \right) $$

**Figure 5:**
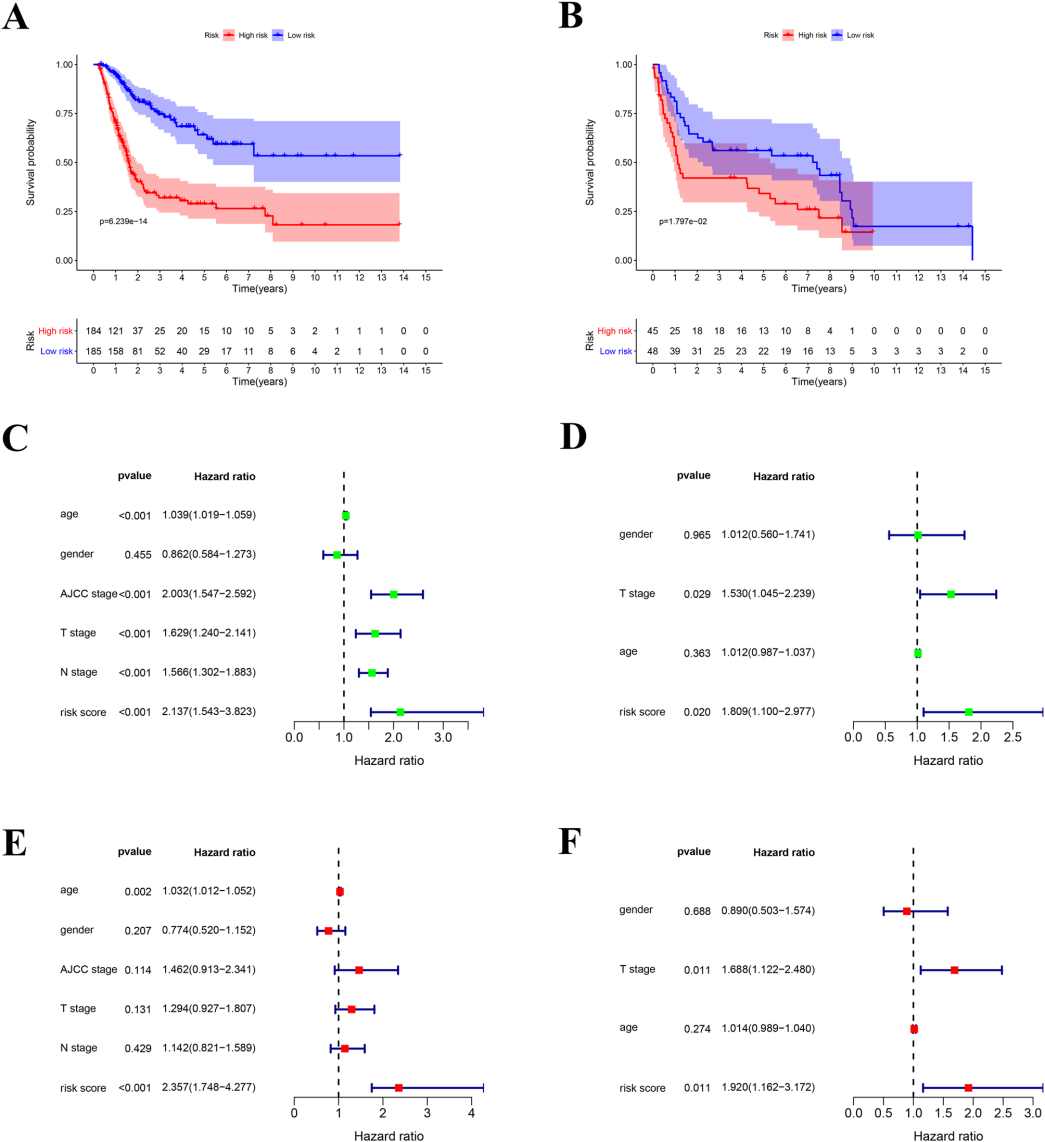
IRGPI and validation model for outcome prediction and relationship with clinical features: (**A**) patients in high-risk group suffered shorter overall survival in IRGPI. (**B**) The forest plot of univariate analyses of risk score with clinical features in IRGPI. (**C**) The forest plot of multivariate analyses of risk score with clinical features in IRGPI. (**D**) Patients in high-risk group suffered shorter overall survival in validation model. (**E**) The forest plot of univariate analyses of risk score with clinical features in validation model. (**F**) The forest plot of multivariate analyses of risk score with clinical features in validation model.

The formula was as follows: [expression level of ADIPOQ* 0.081574] + [expression level of AHNAK* 0.012963] + [expression level of CALR* 0.001428] + [expression level of CMTM8* 0.035383] + [expression level of EDNRA* 0.084329] + [expression level of MMP9* 0.001393] + [expression level of NAMPT* 0.022201] + [expression level of PPY* 0.455283] + [expression level of PROK1* 0.149154] + [expression level of RAC3* 0.024755] + [expression level of RBP7* 0.015126] + [expression level of SLIT2*  − 0.31961] + [expression level of STAT1*  − 0.0065]. We classified patients into high and low risk score groups based on the median riskscore as the cut-off, the survival was analyzed by KM curve. In TCGA prognostic model, high risk score group had worse OS compared to the low riskscore group, *p* < 0.001 (Fig. 5A). In the validation model, high risk score group had worse OS compared to the low riskscore group, *p* < 0.001 (Fig. 5B).

### Clinical application of the prognostic index

The relationship between the prognostic index and clinicopathological features was analysed, including age, sex, American Joint Committee on Cancer (AJCC) stage, tumour stage and lymph node metastasis. Univariate analyses demonstrated that older age (hazard ratio [HR]2.003; 95% CI 1.547–2.592;* p* < 0.001), advanced AJCC stage (HR1.039; 95% CI 1.019–1.059; *p* < 0.001), high T stage (HR 1.629; 95% CI 1.240–2.141; *p* < 0.001), high N stage (HR 1.566; 95% CI 1.302–1.883; *p* < 0.001) and high risk score (HR 1.039; 95% CI 1.027–1.051; *p* < 0.001) were significant risk factors for poor outcome (Fig. 5C). Multivariate analysis indicated that a high risk score (HR 1.809; 95% CI 1.100–2.977; *p* < 0.001) and high T stage (HR 1.530; 95% CI 1.045–2.239; *p* < 0.001) were independently associated with worse OS (Fig. 5D). Of interest, for individual IRGs, our data also showed a close correlation with clinicopathological features (Supplementary material 7–9. In validation prognostic model, univariate analyses showed that older age (HR = 1.032; 95% CI = 1.012–1.052; *p* < 0.001) and high risk score (HR = 2.357; 95% CI = 1.748–4.277; *p* < 0.001) were significant risk factors for poor prognosis (Fig. 5E). Multivariate analysis indicated that a high risk score (HR 1.920; 95% CI 1.162–3.172; *p* = 0.011) and high T stage (HR 1.688; 95% CI 1.122–2.480; *p* = 0.011) were found to be independently associated with worse OS (Fig. 5F).

### Verify the accuracy of the prognostic model

**Figure 6:**
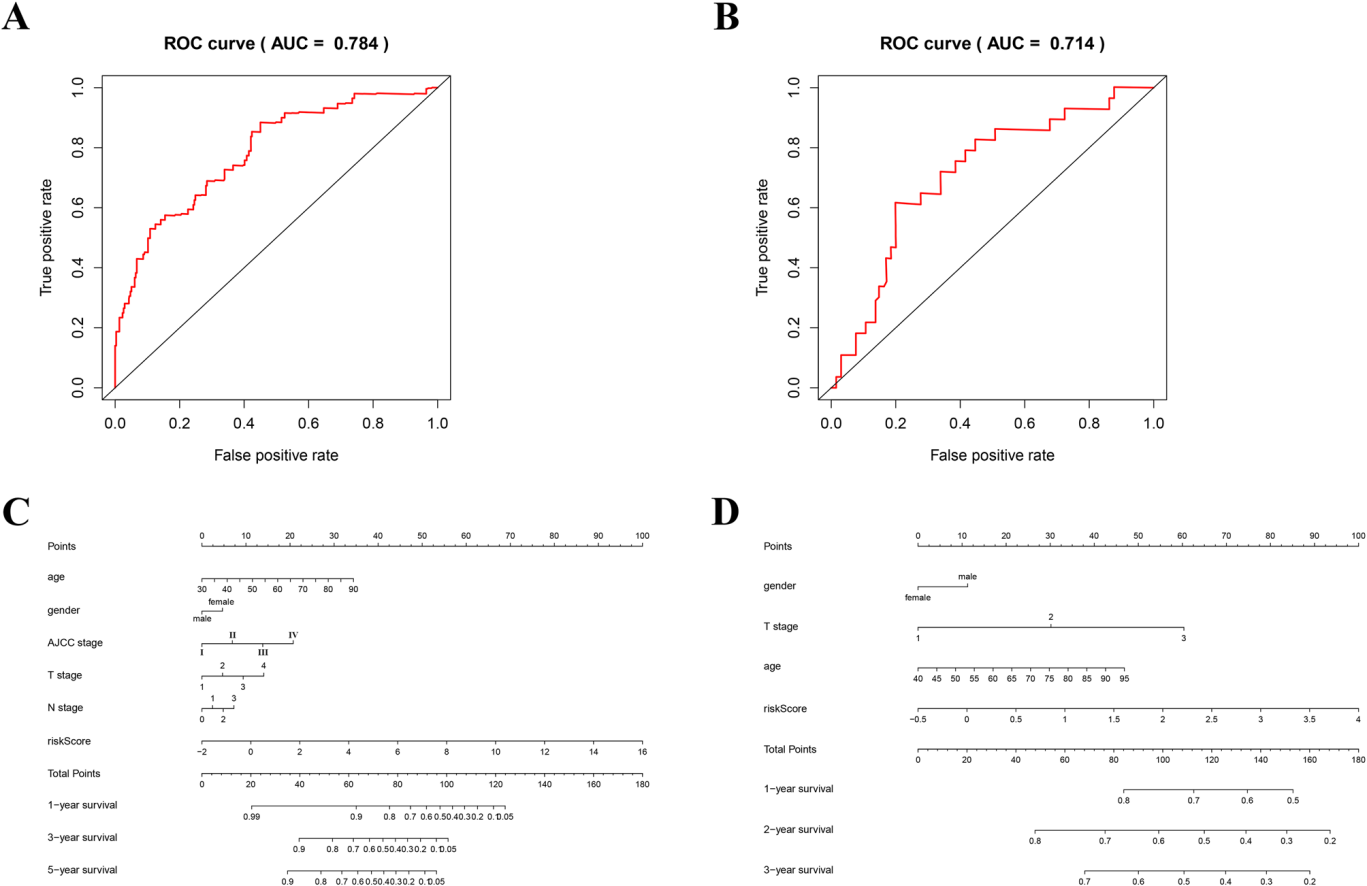
Verify the accuracy of IRGPI and validation model: (**A**) The ROC curve validation of prognostic value of IRGPI. (**B**) The ROC curve validation of prognostic value of validation model. (**C**) The nomogram of IRGPI. (**D**) The nomogram of validation model.

In order to further verify the accuracy of the prognostic model, we constructed the diagram and ROC curve respectively. The ROC curve analysis of TCGA prognostic model was showed in Fig. 6A. The TCGA prognostic model of nomogram was showed in Fig. 6C, and the C-index was 0.734. The ROC curve analysis of GSE31684 validated prognostic model was showed in Fig. 6B. The GSE31684 validated prognostic model of nomogram was showed in Fig. 6D, and the C-index was 0.711.

### Prognostic index and immune cell infiltration correlation analysis

**Figure 7:**
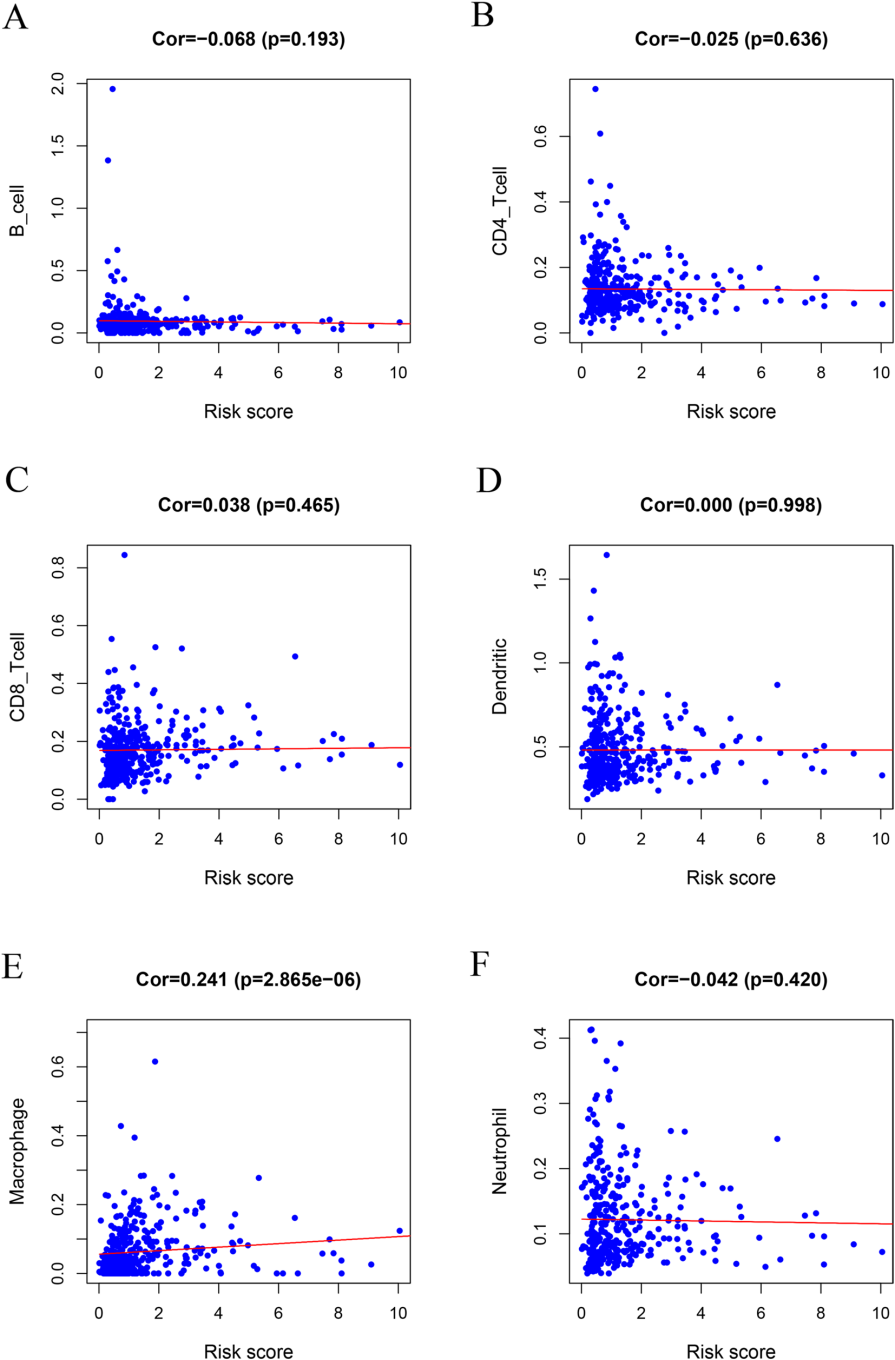
Relationships between the immune-related prognostic index and infiltration of six types of immune cells: (**A**) B cells; (**B**) CD4 T cells; (**C**) CD8 T cells; (**D**) dendritic cells; (**E**) macrophages; and (**F**) neutrophils.

Among six immune cell subtypes, including B cells, CD4 cells, CD8 cells, dendritic cells, macrophages and neutrophils, a high infiltration level of macrophages was observed in high-risk patients (Cor = 0.241, *p* < 0.001) (Fig. 7).

### Regulatory mechanisms of survival-associated IRGs

OGN, ELN, ANXA6, ILK and TGFB3 were identified as hub survival-associated IRGs in the network. EBF1, WWTR1, GATA6, MYH11, and MEF2C were involved in the transcriptional regulation of these survival-associated hub IRGs (Supplementary material 10).

### Analysis and validation of gene expression

**Figure 8:**
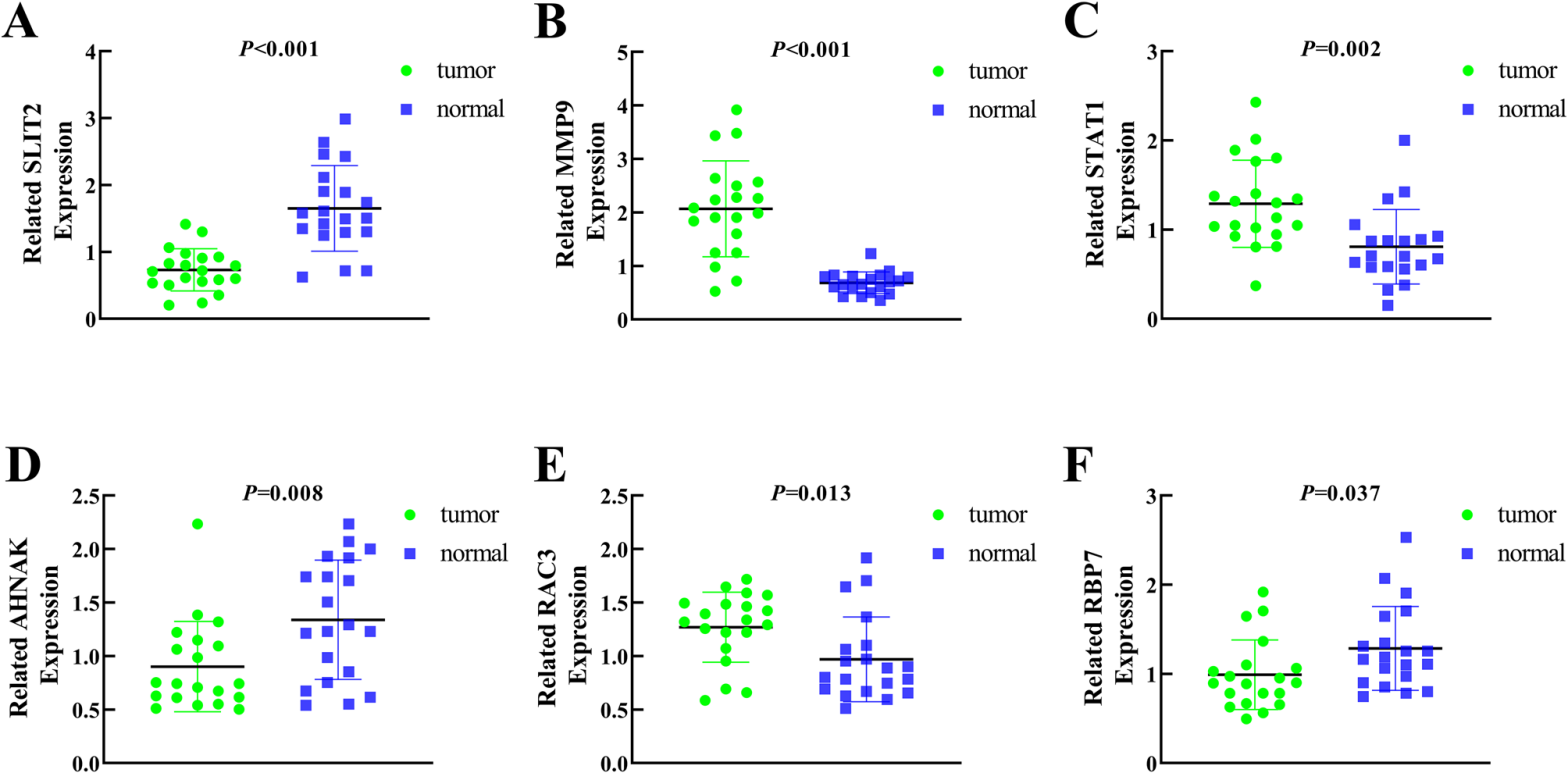
Related expression levels of relevant key genes. (**A**) SLIT2; (**B**) MMP9; (**C**) STAT1; (**D**) AHNAK; (**E**) RAC3; (**F**) RBP7.

To further validate the expression of relevant key genes in the prognostic model, we randomly selected 6 genes to measure the expression level in BC tissue and adjacent normal tissues. We found that the expression is consistent with the TCGA database and GSE31684 (Fig. 8).

## Discussion

The host immune system is well acknowledged to play a pivotal role in tumour initiation and development^13^. Immune cells, an important component of the tumour microenvironment, have shown promise as clinical biomarkers for a variety of malignant tumours^7,8,14^. Previous studies have conducted in-depth investigations of immune-related genes (IRGs) in several cancer types, including gastric cancer^15,16^, pancreatic cancer^17^, thyroid cancer^18^, and non-small-cell lung cancer^19^. However, few studies have explored the role of IRGs in and their predictive value for clinical outcomes in bladder cancer. In the present study, we aimed to construct an immunogenomic prognostic signature based on survival-related IRGs and investigate prognostic biomarkers and potential clinical targeted therapies in bladder cancer.

In the present study, we identified 54 differentially expressed survival-associated IRGs. RAC, a type of Rho-GTPase, is composed of three proteins named RAC1, RAC2, and RAC3^20^. RAC3 has been proven to participate in cell migration, adhesion and apoptosis, playing an important role in breast cancer and lung adenocarcinoma^21–25^. In the study of Walker et al.^22^ RAC3 promoted cancer cell migration, and overexpression of RAC3 in ER-positive breast cancer was correlated with decreased recurrence-free survival. In a recent study, RAC3 was identified to be correlated with the prognosis of prostate cancer^26^. Endothelin A receptor (EDNRA), a G-protein-coupled receptor for endothelin, is widely expressed on vascular smooth muscle cells^27^. ENDRA was strongly correlated with worse outcomes in ovarian cancer^28^. Additionally, Laurberg et al.^29^ reported that high expression of ENDRA was correlated with decreased cancer-specific survival and poor outcome in patients with advanced bladder cancer. AHNAK, a type of desmoyokin, has been shown to be involved in a series of cellular processes, including malignant migration and invasion^30^. Opinions vary regarding the role of AHNAK in tumour progression and outcome prediction. In a recent study by Chen et al.^31^ AHNAK was demonstrated to suppress tumour proliferation and invasion in triple-negative breast cancer. Moreover, AHNAK acts as a tumour suppressor, and down-regulation of AHNAK was independently associated with poor outcome in glioma^32^. Nevertheless, up-regulation of AHNAK was correlated with poor outcome in pancreatic cancer^33^.

In the KEGG pathway analysis, “MAPK signalling pathway”, “IL-17 signalling pathway”, “cytokine-cytokine interaction receptor”, “neuroactive ligand-receptor interaction” and “viral protein interaction with cytokine and cytokine receptor” were identified as the top 5 enriched pathways. Mitogen-activated protein kinases (MAPKs) participate in a series of biological functions, including cell proliferation, angiogenesis, and metastasis of malignant tumours, including bladder cancer^34–36^. For instance, in a recent study by Zhao et al.^36^ benzidine was demonstrated to enhance the proliferation of bladder cells via the MAPK/AP-1 signalling pathway. To date, up-regulation of pyruvate kinase M2 has been shown to promote bladder cancer metastasis by facilitating cell proliferation, invasion and migration via the MAPK signalling pathway^37^. Moreover, the present study indicated that RAC3 was affected by the MAPK signalling pathway. RAC3 has been demonstrated to promote cell invasion and migration via the p38 MAPK pathway in lung adenocarcinoma^25^. To the best of our knowledge, no studies have reported the correlation between RAC3 and the MAPK signalling pathway in bladder cancer. Thus, the molecular mechanism of MAPK signalling pathway regulation is worthy of exploration and study.

The present study constructed a prognostic index for bladder cancer for the first time. Our prognostic index showed promising clinical feasibility. Our findings indicated that patients with high risk scores showed more advanced stages and larger tumour sizes than patients with low risk scores. Of interest, for individual IRGs, our data also showed a close correlation with clinicopathological features. For instance, up-regulation of ADIPOQ, AGTR1, AHNAK, ENDRA, RBP7 and SLIT2 was correlated with larger tumour size and more advanced tumour stage. RLN2 and NAMPT were correlated with sex.

Moreover, a combinatory analysis of immune cell infiltration and prognostic index was performed to investigate the tumour immune microenvironment. In the present study, the prognostic index had a significantly positive correlation with the infiltration of macrophages. This result indicated that a higher infiltration level of macrophages was probably observed in high-risk patients. With the influence of chemoattractants and other stimuli, circulating monocytes are recruited into the tumour site and differentiate into tumour-associated macrophages (TAMs)^38^. TAMs can be categorized into the anti-tumour M1 phenotype and the pro-tumorigenic M2 phenotype, and the M2 phenotype accounts for the majority of TAMs^39^. Notably, M2-phenotype TAMs have been correlated with poor clinical outcomes in pancreatic cancer, breast cancer and lung cancer^40,41^. CD204^+^ macrophages, in other words, M2 macrophages, were significantly associated with larger tumour size, more advanced tumour stage, higher tumour grade and more nodal metastasis in bladder cancer^42^. Coincidentally, recent studies have demonstrated that M2-phenotype TAMs are the overwhelming immune cell type in the microenvironment of bladder cancer^43^. Thus, M2 macrophages can be used as a promising prognostic index and potential target for immunotherapy in bladder cancer.

We further investigated the potential mechanism of survival-associated IRGs and the clinical significance via analysis of the expression profiles of transcription factors. OGN, ELN, ANXA6, ILK and TGFB3 were identified as hub IRGs in the network. Osteoinductive factor (OGN) has been demonstrated to reduce cell proliferation and inhibit invasion in colorectal cancer^44^. A recent study reported that integrin-linked kinase (ILK) could promote cell proliferation and migration in non-small-cell lung cancer^45^. Moreover, transforming growth factor (TGF)-B3 has been widely accepted as a crucial mediator of tumour progression^46–48^. MYH11, which encodes myosin heavy chain 11, a smooth muscle myosin protein, is closely associated with the composition of the fusion gene CBFB/MYH11 and participates in the initiation of acute myeloid leukaemia^49^. In general, previous studies have provided a limited understanding of the regulatory effects of IRGs on bladder cancer outcomes. The immunogenomic mechanisms of IRGs in bladder cancer are worthy of deep exploration.

## Limitations

The present study still had several limitations. First, our prognostic index was based on gene expression data provided by TCGA, and the high price and long testing time might limit the application of our prognostic index in clinical practice. Second, because of limited clinical data, the present study failed to designate subgroups, and for patients receiving immunotherapy, our study might not accurately reflect the immune cell infiltration status. Moreover, the reliability of IRGs and their clinical prognostic value require further investigation for verification.

## Conclusion

To the best of our knowledge, this is the first study to perform an immunogenomic landscape analysis and construct an IRG-related prognostic index in bladder cancer. In addition, our study revealed that macrophage infiltration was positively correlated with bladder cancer outcome, providing a more comprehensive understanding of the immune response in the tumour microenvironment and promising immunotherapeutic targets for clinical practice.

## Methods

### Data acquisition and processing

Transcriptome RNA-sequencing and clinical data from bladder cancer patients were obtained from the TCGA data portal (https://portal.gdc.cancer.gov/). A total of 411 bladder cancer tissues and 19 normal bladder tissues were included in the present study. The immune-related gene list was downloaded from the Immunology Database and Analysis Portal (ImmPort) database^50^.

### Identification of DEGs, DEIRGs and survival-associated IRGs

The differentially expressed genes (DEGs) were identified by using the edgeR package in the R language (https://bioconductor.org/packages/edgeR/) to further analyse the data. A |log2 fold change (FC)|> 2.0 and false discovery rate (FDR) adjusted to a P value < 0.01 were set as the thresholds^51^. In addition, volcano maps and heat maps of the DEGs were produced by using the gplots and heatmap packages in the edgeR package. By compared the immune-related gene lists, we obtained the DEIRGs. Survival-associated IRGs were selected by univariate Cox analysis, which was conducted using the R software survival package.

### Functional enrichment analysis

To understand the underlying biological mechanisms of the IRGs, GO annotation and KEGG pathway analyses were conducted by the DAVID (Database for Annotation, Visualization, and Integrated discovery; https://david.ncifcrf.gov/) online tool^52^ and clusterProfiler, which is an R package for functional classification and enrichment of gene clusters using hypergeometric distribution. The GO plot package of the R software was used to display the results of the GO and KEGG analyses. GO and KEGG enrichment analyses were based on the threshold of *p* < 0.01.

### Development of the immune-related gene prognostic index (IRGPI) and validation model

Prognostic risk score was obtained for all patients by univariate COX regression analysis and lasso-penalized Cox regression. Cox regression analysis was tested by Akaike Information Criterion (AIC) to identify the predictive model with the best explanatory and informative efficacy^53^. Patients were classified into high risk score group and low risk score group by median risk score. To further verify the feasibility of the prognostic model, we also divided GSE31684 patients into two groups according to the median risk score. The survival of the two groups of patients was analyzed by KM curve.

### Relationship between IRGPI and immune cell infiltration

The TIMER online database analyses and visualizes the abundances of tumour-infiltrating immune cells^54^. TIMER was used to reanalyse gene expression data from TCGA, which included 10,897 samples across 32 cancer types, to estimate the abundance of six subtypes of tumour-infiltrating immune cells, including CD4 cells, CD8 cells, B cells, macrophages, dendritic cells and neutrophils. TIMER can be easily used to determine the relationship between immune cell infiltration and other parameters. We downloaded the immune infiltrate levels of bladder cancer patients and calculated associations between the IRGPI and immune cell infiltration.

### Regulatory mechanisms of survival-associated IRGs

The Cistrome Cancer database is a resource for experimental and computational cancer biology research and contains a total of 318 transcription factors (TFs). To explore how TFs regulate the clinically relevant IRGs, we screened out clinically relevant TFs to construct the regulatory network of relevant IRGs and potential TFs.

### Quantitative real-time PCR (qRT-PCR)

Total RNA was obtained from 20 patients with breast cancer using TRIzol reagent (Invitrogen), and then reverse transcribed with the First Strand cDNA synthesis kit (New England Biolabs (Beijing) LTD., China). We performed amplifications with a SYBR Green PCR kit (Applied Biological Materials, Canada) according to the manufacturer’s instructions on Applied Biosystems 7500Real-Time PCR System (Applied Biosystems, USA). The expression of RNA was normalized against GAPDH using the 2-ΔΔCt method. The PCR primers used are shown in Supplementary material 11. Three separate experiments were performed.

### Statistical analysis

We performed survival analysis for patients with the prognostic model by using the “survival” package in R. Survival curves were generated using the Kaplan–Meier method and the log-rank test to compare the difference between the two groups. The AUC of the survival ROC curve was calculated via the survival ROC R software package to validate the performance of the prognostic signature^55^. *p* values of < 0.05 were considered statistically significant.

## Supplementary information

Supplementary Information 1.

Supplementary Information 2.

Supplementary Information 3.

Supplementary Information 4.

Supplementary Information 5.

Supplementary Information 6.

Supplementary Information 7.

Supplementary Information 8.

Supplementary Information 9.

Supplementary Information 10.

Supplementary Information 11.

Supplementary Information 12.
