# Supplementary figures and images for: Identification of immune-related genes as prognostic factors in bladder cancer
Source: Sci Rep. 2020 Nov 12;10:19695. doi: 10.1038/s41598-020-76688-w (PMC7661532; doi:10.1038/s41598-020-76688-w)

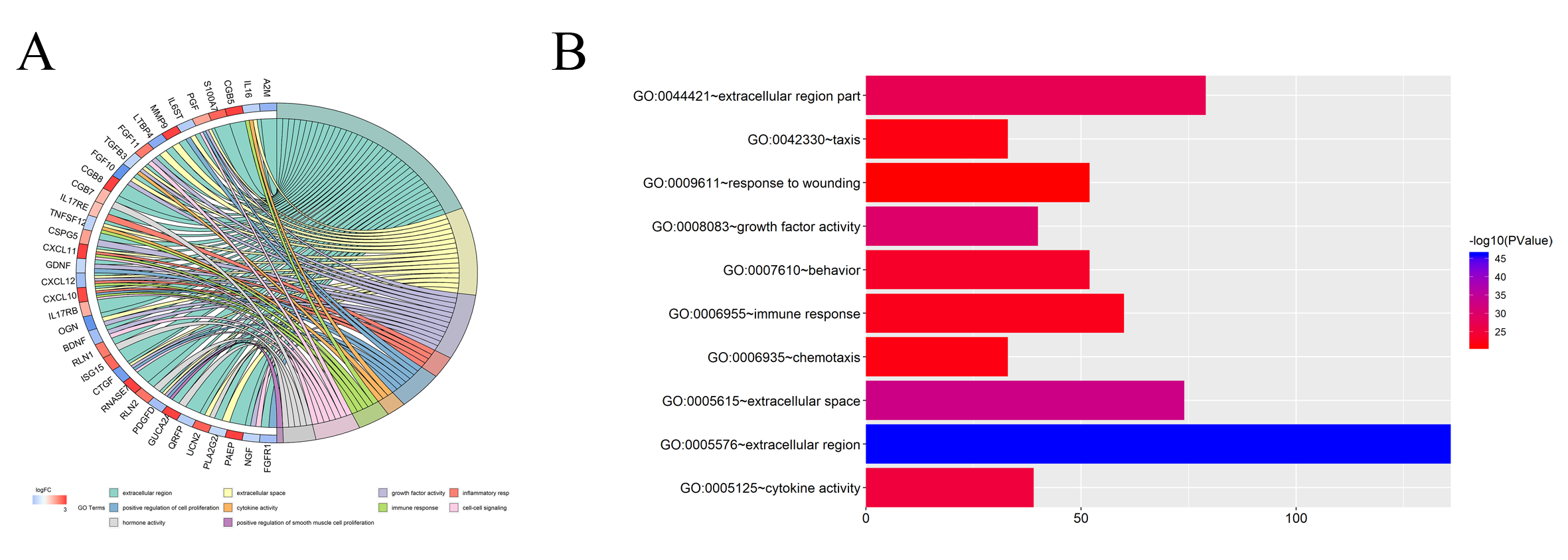

Supplement: Supplementary file 1 — Supplementary Information 1. [file 41598_2020_76688_MOESM1_ESM.tif]

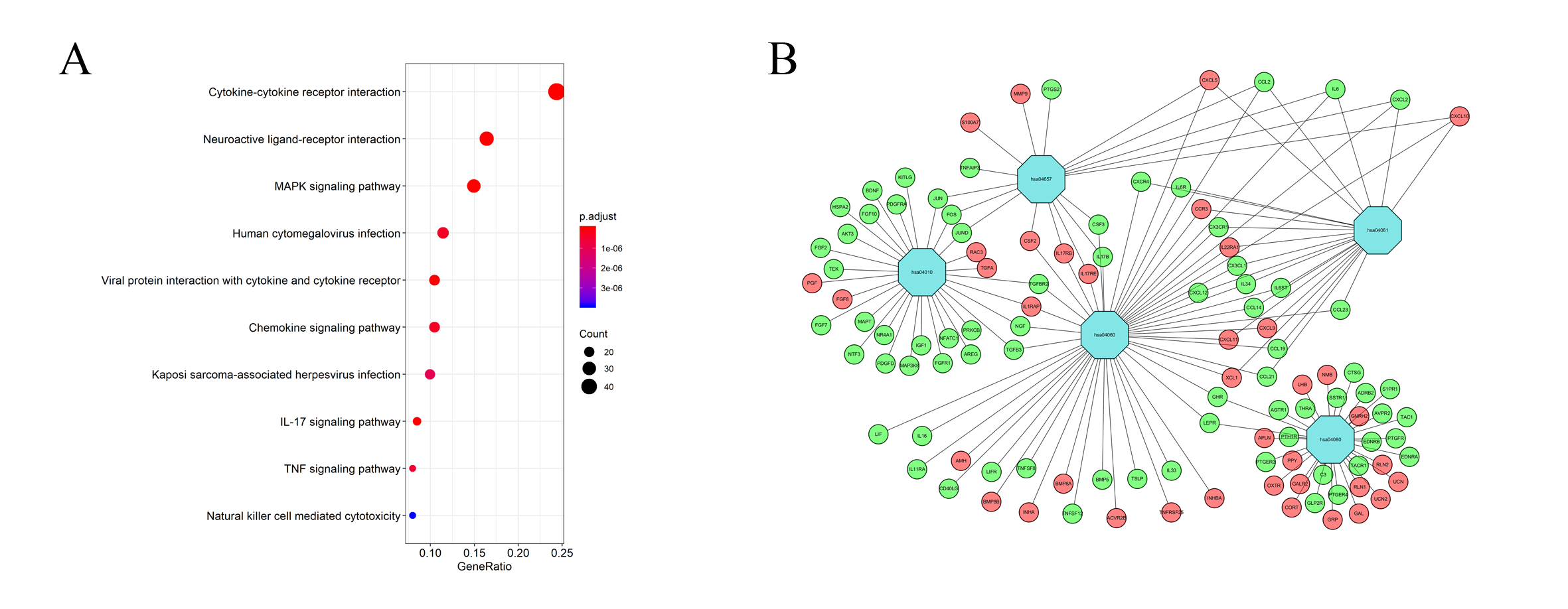

Supplement: Supplementary file 3 — Supplementary Information 3. [file 41598_2020_76688_MOESM3_ESM.tif]

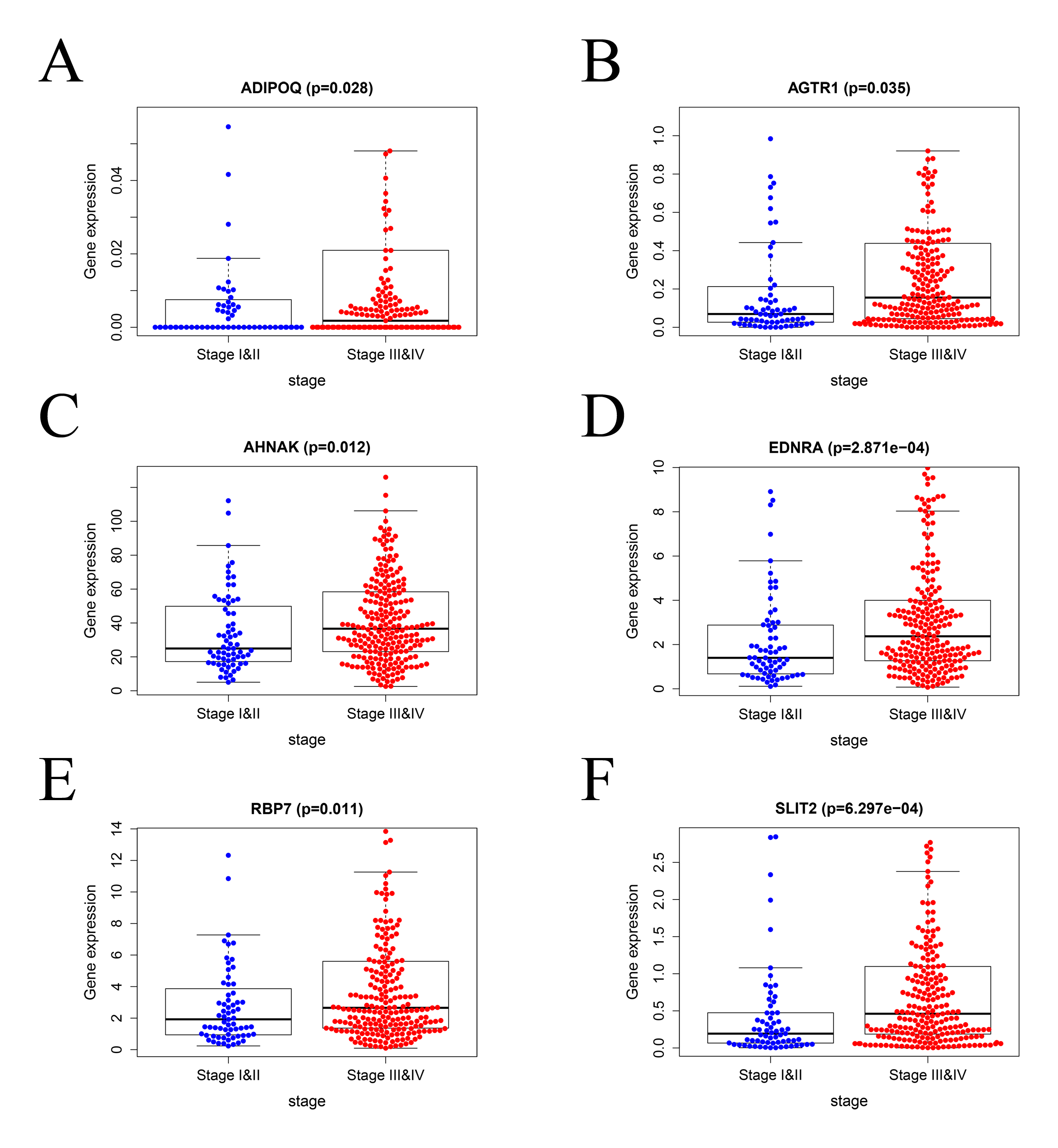

Supplement: Supplementary file 7 — Supplementary Information 7. [file 41598_2020_76688_MOESM7_ESM.tif]

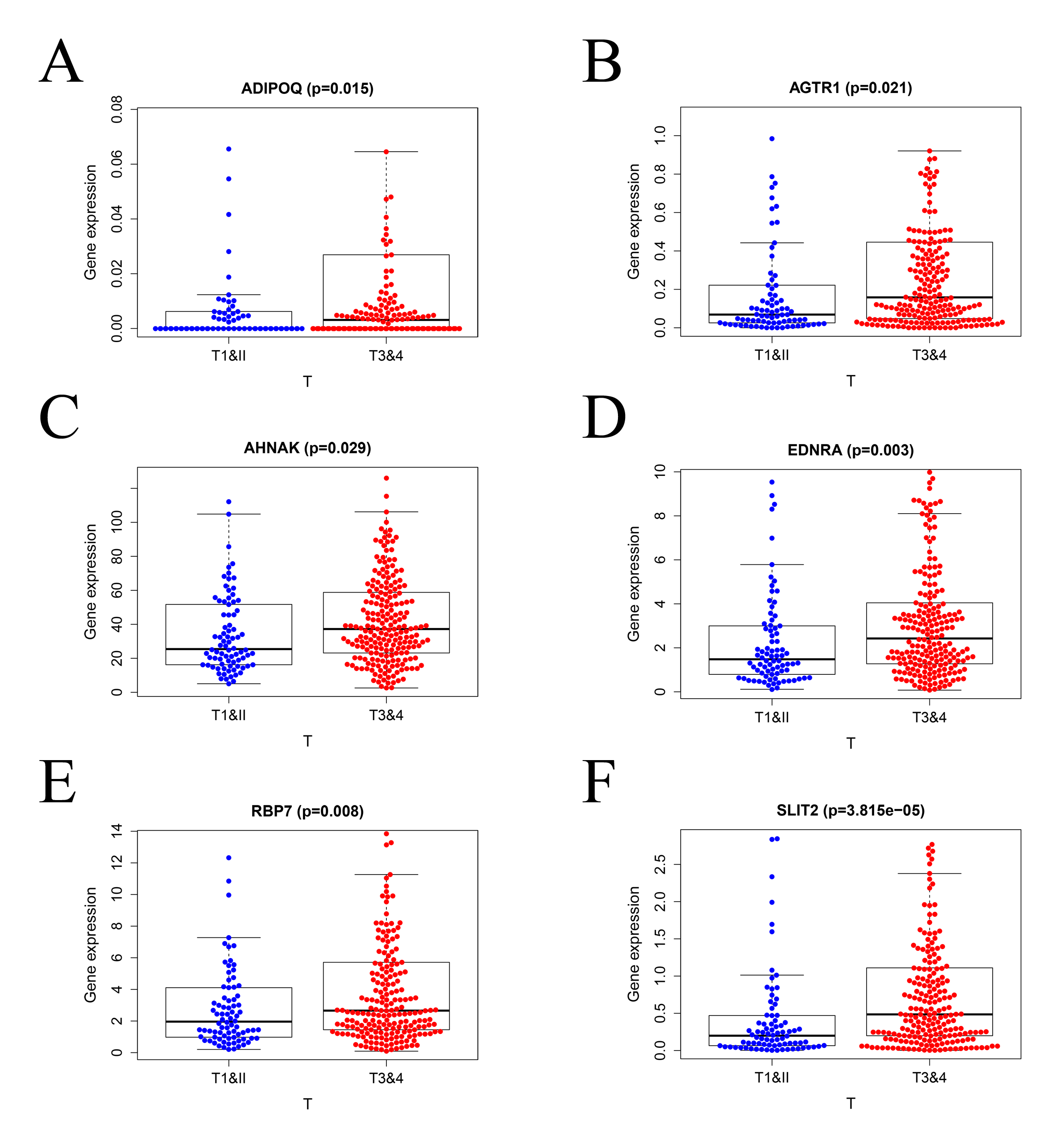

Supplement: Supplementary file 8 — Supplementary Information 8. [file 41598_2020_76688_MOESM8_ESM.tif]

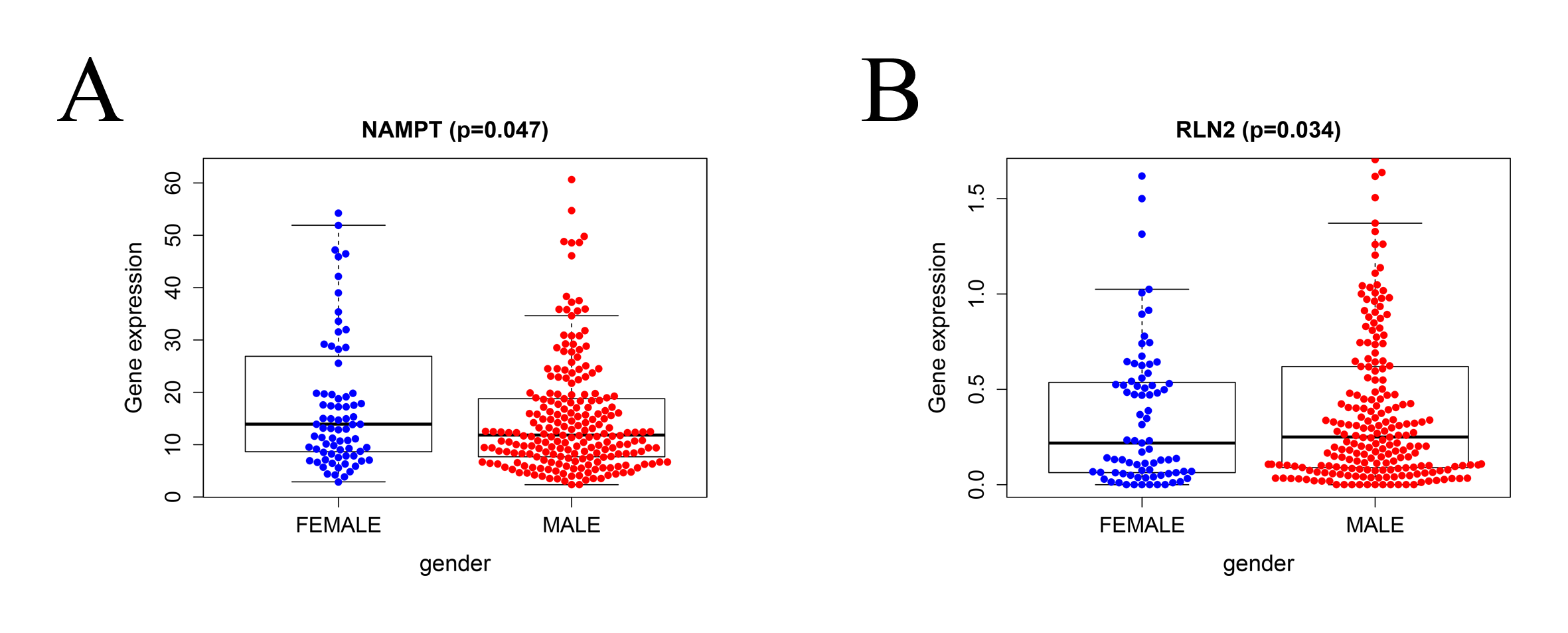

Supplement: Supplementary file 9 — Supplementary Information 9. [file 41598_2020_76688_MOESM9_ESM.tif]

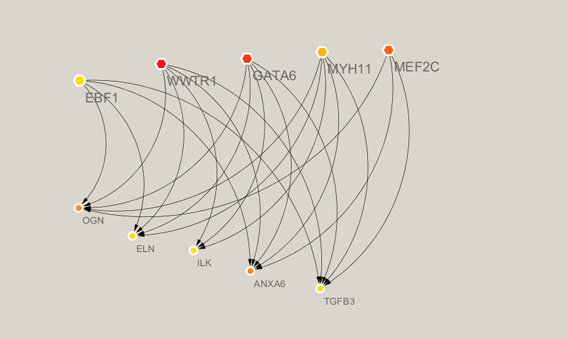

Supplement: Supplementary file 10 — Supplementary Information 10. [file 41598_2020_76688_MOESM10_ESM.tif]
