# Supplementary material for: Identification of immune-related genes as prognostic factors in bladder cancer
Source: Sci Rep. 2020 Nov 12;10:19695. doi: 10.1038/s41598-020-76688-w (PMC7661532; doi:10.1038/s41598-020-76688-w)
Supplement: Supplementary file 2 — Supplementary Information 2. [file 41598_2020_76688_MOESM2_ESM.pdf]

Supplementary material 2. GO biological process categories of differentially expressed genes.

Term	GeneRatio	Genes	*P* Value
extracellular region	125/259	A2M, IL16, CGB5, S100A7, PGF, IL6ST, MMP9, LTBP4, FGF11, TGFB3, FGF10, CGB8, CGB7, IL17RE, TNFSF12, CSPG5, CXCL11, GDNF, CXCL12, CXCL10, IL17RB, OGN, BDNF, RLN1, ISG15, CTGF, RNASE7, RLN2, PDGFD, GUCA2A, QRFP, UCN2, PLA2G2A, PAEP, NGF, FGFR1, PPY, CCL2, ELN, GAST, DEFB126, OAS1, CX3CL1, IL33, ESM1, CALR, IL34, MDK, FAM19A3, GREM2, CHIT1, ANGPTL7, LIF, IL17B, ALB, TEK, CKLF, SPP1, NTF3, GNRH2, TINAGL1, S100B, TGFBR3, ADM2, XCL1, CORT, LHB, BMP5, PLAU, BMP8A, NRP2, FGF8, FGF7, LEPR, SEMA3E, IL1RAP, SEMA3A, LBP, APLN, NRG2, FGF2, GHR, CYR61, ARTN, INHA, IL6R, GAL, SLIT2, C8G, PROC, GRP, TOR2A, INHBA, AMH, PTGDS, PROK1, CTSG, CSF3, CSF2, WFIKKN1, CXCL5, C3, TNFRSF25, CXCL2, CXCL9, TAC1, KITLG, NMB, MIF, CCL23, CCL21, PTX3, THBS1, IL6, LCN12, RNASE2, IGF1, CCL19, PTGFR, ADIPOQ, CCL14, DKK1, TFRC, HBEGF, CMA1	9.46E-47
extracellular space	99/259	IL16, PGF, IL6ST, MMP9, LTBP4, TGFB3, FGF10, TNFSF12, CXCL11, CXCL12, CXCL10, OGN, CTGF, PDGFD, UCN2, CD40LG, CST4, PLA2G2A, STC1, PPY, CCL2, GAST, CX3CL1, IL33, CALR, IL34, GREM2, CHIT1, LIF, IL17B, ALB, CKLF, ANGPTL1, ANGPTL2, SPP1, UCN, S100A11, TINAGL1, S100B, TGFBR3, AREG, XCL1, CORT, LHB, BMP5, PLAU, BMP8B, BMP8A, NAMPT, FGF8, VGF, SEMA3G, HMOX1, SEMA3E, TGFA, SEMA3A, LBP, NRG2, FGF2, APLN, GHR, ACTA1, ARTN, IL6R, GAL, SLIT2, PROC, GRP, AMH, PTGDS, SEMA4F, ULBP2, CTSG, CSF3, CSF2, CXCL5, C3, CXCL2, CXCL9, TAC1, KITLG, MIF, CCL23, CCL21, THBS1, PTX3, CMTM1, IL6, CCL19, IGF1, TMPRSS6, ADIPOQ, TNFSF8, TSLP, CCL14, DKK1, TFRC, CMTM8, HBEGF	1.09E-33
growth factor activity	39/259	CSF3, CSF2, FGF8, FGF7, PGF, TGFB3, FGF11, KITLG, FGF10, CSPG5, VGF, IL34, GDNF, CXCL12, MDK, LIF, OGN, BDNF, TYMP, CTGF, TGFA, PDGFD, NRG2, FGF2, IL6, NTF3, ARTN, IGF1, INHA, INHBA, AMH, DKK1, PROK1,	1.46E-30

		HBEGF, AREG, BMP8B, BMP5, NGF, BMP8A	
extracellular region part	40/259	CCL2, CXCL5, PTGS2, TNFRSF25, C3, TACR1, CXCL2, TLR2, CXCL9, TAC1, IL17RE, IL34, CXCL11, CXCL12, CXCL10, MIF, SLC11A1, FOS, IL17B, CCL23, CXCR4, CCL21, IL1RAP, NFATC4, PTX3, THBS1, SPP1, NFKBIZ, IL6, UCN, PTGER3, OLR1, CCL19, GAL, PTGFR, NCR3, CCL14, CD40LG, CCR3, TNFAIP3	4.26E-28
cytokine activity	41/259	CSF3, NAMPT, CSF2, AVPR2, FGFR1, FGF8, FGF7, CXCL5, IL6ST, PGF, PTH1R, NMB, ESM1, IL34, CALR, GDNF, CXCL10, LIF, EDNRB, CTGF, ILK, TGFA, GLP2R, PDGFD, THBS1, APLN, FGF2, AR, IL6, NTF3, PDF, TGFBR2, LIFR, IGF1, BIRC5, IL6R, PTGFR, CCL14, S100B, PROK1, PDGFRA, HBEGF, AREG	6.44E-26
behavior	29/259	CSF3, CSF2, NAMPT, IL16, CXCL9, TGFB3, KITLG, IL33, TNFSF12, IL34, GREM2, MIF, LIF, IL17B, FGF2, SPP1, IL6, CMTM1, INHA, ADIPOQ, TNFSF8, TSLP, INHBA, CD40LG, CMTM8, AREG, BMP8B, BMP5, BMP8A	3.90E-24
immune response	40/259	CSF3, CSF2, CCL2, AQP9, IL16, CXCL5, C3, TNFRSF25, CXCL2, TLR2, CXCL9, OAS1, TNFSF12, CX3CL1, CXCL11, CXCL12, CHIT1, CXCL10, LIF, SLC11A1, IL17B, CCL23, CCL21, IL1RAP, FCGR3A, THBS1, APLN, IL6, PTGER4, CCL19, TINAGL1, CD1E, NCR3, TNFSF8, CCL14, CD40LG, TGFBR3, CMA1, CD79B, CTSG	6.18E-23
taxis	32/259	NAMPT, PPY, CXCL5, PGF, CXCL9, FGF11, TAC1, NMB, CXCL11, CXCL10, BDNF, IL17B, CCL23, CCL21, CTGF, TEK, CYR61, AR, NTF3, BST2, INHA, TNFSF8, INHBA, AMH, ADRB2, SSTR1, SEMA4F, AREG, GRAP2, XCL1, LHB, NGF	2.36E-22
chemotaxis	19/259	PPY, GNRH2, IGF1, GAST, CGB7, NMB, INHA, VGF, ADIPOQ, AMH, INHBA, UCN2, RLN1, RLN2, STC1, ADM2, GUCA2A, LHB, APLN	2.36E-22
response to wounding	15/259	NAMPT, IL6, PTGS2, TGFBR2, IGF1, IL6R, NR4A3, STAT1, S1PR1, JUN, HMOX1, ABCC4, HBEGF, PDGFD, THBS1, FGF2	1.21E-21
