# Supplementary material for: Identification of immune-related genes as prognostic factors in bladder cancer
Source: Sci Rep. 2020 Nov 12;10:19695. doi: 10.1038/s41598-020-76688-w (PMC7661532; doi:10.1038/s41598-020-76688-w)
Supplement: Supplementary file 4 — Supplementary Information 4. [file 41598_2020_76688_MOESM4_ESM.pdf]

Supplementary material 4. KEGG pathway categories of differentially expressed genes.

Description	GeneRatio	Gene ID	*P* value
Cytokine-cytokine receptor interaction	49/201	CXCL10/CXCL9/CXCL5/CXCL11/CXCL12/CXCL2/XCL1/IL6/CD40LG/CCL14/CCL19/CCR3/CCL2/CCL21/CCL23/CXCR4/CX3CL1/CX3CR1/AMH/BMP5/BMP8A/BMP8B/CSF2/CSF3/IL16/IL17B/IL33/IL34/IL6ST/INHA/INHBA/LIF/NGF/TGFB3/TNFSF12/TNFSF8/TSLP/ACVR2B/GHR/IL11RA/IL17RB/IL17RE/IL1RAP/IL22RA1/IL6R/LEPR/LIFR/TGFBR2/TNFRSF25	5.51E-25
Viral protein interaction with cytokine and cytokine receptor	21/201	CXCL10/CXCL9/CXCL5/CXCL11/CXCL12/CXCL2/XCL1/IL6/CCL14/CCL19/CCR3/CCL2/CCL21/CCL23/CXCR4/CX3CL1/CX3CR1/IL34/IL6ST/IL22RA1/IL6R	4.40E-12
Neuroactive ligand-receptor interaction	33/201	CTSG/C3/EDNRA/EDNRB/APLN/CORT/GAL/GNRH2/GRP/LHB/NMB/PPY/RLN1/RLN2/TAC1/UCN/UCN2/ADRB2/AGTR1/AVPR2/GALR2/GHR/GLP2R/LEPR/OXTR/PTGER3/PTGER4/PTGFR/PTH1R/S1PR1/SSTR1/TACR1/THRA	1.13E-09
MAPK signaling pathway	30/201	HSPA2/MAPT/JUN/PDGFRA/FGF2/JUND/RAC3/NFATC1/FOS/AKT3/PRKCB/FGF10/AREG/BDNF/FGF7/FGF8/IGF1/KITLG/NGF/NTF3/PDGFD/PGF/TGFA/TGFB3/FGFR1/IL1RAP/NR4A1/TEK/TGFBR2/MAP3K8	2.90E-09
IL-17 signaling pathway	17/201	CXCL10/CXCL5/CXCL2/S100A7/IL6/MMP9/TNFAIP3/JUN/JUND/PTGS2/CCL2/FOS/CSF2/CSF3/IL17B/IL17RB/IL17RE	5.46E-09
