# Supplementary material for: Identification of immune-related genes as prognostic factors in bladder cancer
Source: Sci Rep. 2020 Nov 12;10:19695. doi: 10.1038/s41598-020-76688-w (PMC7661532; doi:10.1038/s41598-020-76688-w)
Supplement: Supplementary file 5 — Supplementary Information 5. [file 41598_2020_76688_MOESM5_ESM.pdf]

**Supplementary material 5. GO biological process categories of differentially expressed immune-related genes (DEIRGs).**

| Term | Genes | *P* Value |
|---|---|---|
| extracellular region | A2M, IL16, CGB5, S100A7, PGF, IL6ST, MMP9, LTBP4, FGF11, TGFB3, FGF10, CGB8, CGB7, IL17RE, TNFSF12, CSPG5, CXCL11, GDNF, CXCL12, CXCL10, IL17RB, OGN, BDNF, RLN1, ISG15, CTGF, RNASE7, RLN2, PDGFD, GUCA2A, QRFP, UCN2, PLA2G2A, PAEP, NGF, FGFR1, PPY, CCL2, ELN, GAST, DEFB126, OAS1, CX3CL1, IL33, ESM1, CALR, IL34, MDK, FAM19A3, GREM2, CHIT1, ANGPTL7, LIF, IL17B, ALB, TEK, CKLF, SPP1, NTF3, GNRH2, TINAGL1, S100B, TGFBR3, ADM2, XCL1, CORT, LHB, BMP5, PLAU, BMP8A, NRP2, FGF8, FGF7, LEPR, SEMA3E, IL1RAP, SEMA3A, LBP, APLN, NRG2, FGF2, GHR, CYR61, ARTN, INHA, IL6R, GAL, SLIT2, C8G, PROC, GRP, TOR2A, INHBA, AMH, PTGDS, PROK1, CTSG, CSF3, CSF2, WFIKKN1, CXCL5, C3, TNFRSF25, CXCL2, CXCL9, TAC1, KITLG, NMB, MIF, CCL23, CCL21, PTX3, THBS1, IL6, LCN12, RNASE2, IGF1, CCL19, PTGFR, ADIPOQ, CCL14, DKK1, TFRC, HBEGF, CMA1 | 9.46E-47 |
| extracellular space | IL16, PGF, IL6ST, MMP9, LTBP4, TGFB3, FGF10, TNFSF12, CXCL11, CXCL12, CXCL10, OGN, CTGF, PDGFD, UCN2, CD40LG, CST4, PLA2G2A, STC1, PPY, CCL2, GAST, CX3CL1, IL33, CALR, IL34, GREM2, CHIT1, LIF, IL17B, ALB, CKLF, ANGPTL1, ANGPTL2, SPP1, UCN, S100A11, TINAGL1, S100B, TGFBR3, AREG, XCL1, CORT, LHB, BMP5, PLAU, BMP8B, BMP8A, NAMPT, FGF8, VGF, SEMA3G, HMOX1, SEMA3E, TGFA, SEMA3A, LBP, NRG2, FGF2, APLN, GHR, ACTA1, ARTN, IL6R, GAL, SLIT2, PROC, GRP, AMH, PTGDS, SEMA4F, ULBP2, CTSG, CSF3, CSF2, CXCL5, C3, CXCL2, CXCL9, TAC1, KITLG, MIF, CCL23, CCL21, THBS1, PTX3, CMTM1, IL6, CCL19, IGF1, TMPRSS6, ADIPOQ, TNFSF8, TSLP, CCL14, DKK1, TFRC, CMTM8, HBEGF | 1.09E-33 |
| growth factor activity | CSF3, CSF2, FGF8, FGF7, PGF, TGFB3, FGF11, KITLG, FGF10, CSPG5, VGF, IL34, GDNF, CXCL12, MDK, LIF, OGN, BDNF, TYMP, CTGF, TGFA, PDGFD, NRG2, FGF2, IL6, NTF3, ARTN, IGF1, INHA, INHBA, AMH, DKK1, PROK1, HBEGF, AREG, BMP8B, BMP5, NGF, BMP8A | 1.46E-30 |
| extracellular region part | CCL2, CXCL5, PTGS2, TNFRSF25, C3, TACR1, CXCL2, TLR2, CXCL9, TAC1, IL17RE, IL34, CXCL11, CXCL12, CXCL10, MIF, SLC11A1, FOS, IL17B, CCL23, CXCR4, CCL21, IL1RAP, NFATC4, PTX3, THBS1, SPP1, NFKBIZ, IL6, UCN, PTGER3, OLR1, CCL19, GAL, PTGFR, NCR3, CCL14, CD40LG, CCR3, TNFAIP3 | 4.26E-28 |
| cytokine activity | CSF3, NAMPT, CSF2, AVPR2, FGFR1, FGF8, FGF7, CXCL5, IL6ST, PGF, PTH1R, NMB, ESM1, IL34, CALR, GDNF, CXCL10, LIF, EDNRB, CTGF, ILK, TGFA, GLP2R, PDGFD, THBS1, APLN, | 6.44E-26 |

| | | |
|---|---|---|
| | FGF2, AR, IL6, NTF3, PDF, TGFBR2, LIFR, IGF1, BIRC5, IL6R, PTGFR, CCL14, S100B, PROK1, PDGFRA, HBEGF, AREG | |
| behavior | CSF3, CSF2, NAMPT, IL16, CXCL9, TGFB3, KITLG, IL33, TNFSF12, IL34, GREM2, MIF, LIF, IL17B, FGF2, SPP1, IL6, CMTM1, INHA, ADIPOQ, TNFSF8, TSLP, INHBA, CD40LG, CMTM8, AREG, BMP8B, BMP5, BMP8A | 3.90E-24 |
| immune response | CSF3, CSF2, CCL2, AQP9, IL16, CXCL5, C3, TNFRSF25, CXCL2, TLR2, CXCL9, OAS1, TNFSF12, CX3CL1, CXCL11, CXCL12, CHIT1, CXCL10, LIF, SLC11A1, IL17B, CCL23, CCL21, IL1RAP, FCGR3A, THBS1, APLN, IL6, PTGER4, CCL19, TINAGL1, CD1E, NCR3, TNFSF8, CCL14, CD40LG, TGFBR3, CMA1, CD79B, CTSG | 6.18E-23 |
| taxis | NAMPT, PPY, CXCL5, PGF, CXCL9, FGF11, TAC1, NMB, CXCL11, CXCL10, BDNF, IL17B, CCL23, CCL21, CTGF, TEK, CYR61, AR, NTF3, BST2, INHA, TNFSF8, INHBA, AMH, ADRB2, SSTR1, SEMA4F, AREG, GRAP2, XCL1, LHB, NGF | 2.36E-22 |
| chemotaxis | PPY, GNRH2, IGF1, GAST, CGB7, NMB, INHA, VGF, ADIPOQ, AMH, INHBA, UCN2, RLN1, RLN2, STC1, ADM2, GUCA2A, LHB, APLN | 2.36E-22 |
| response to wounding | NAMPT, IL6, PTGS2, TGFBR2, IGF1, IL6R, NR4A3, STAT1, S1PR1, JUN, HMOX1, ABCC4, HBEGF, PDGFD, THBS1, FGF2 | 1.21E-21 |
