# Supplementary material for: Identification of immune-related genes as prognostic factors in bladder cancer
Source: Sci Rep. 2020 Nov 12;10:19695. doi: 10.1038/s41598-020-76688-w (PMC7661532; doi:10.1038/s41598-020-76688-w)
Supplement: Supplementary file 6 — Supplementary Information 6. [file 41598_2020_76688_MOESM6_ESM.pdf]

Supplementary material 6. KEGG pathway categories of differentially expressed immune-related genes (DEIRGs).

Description	GeneRatio	Gene ID	*P* value
MAPK signaling pathway	11/41	IGF1/KITLG/MAP3K8/NFATC1/NGF/PDGFD/PDGFRA/PGF/RAC3/TGFB3/TGFBR2	2.57E-05
Human cytomegalovirus infection	9/41	CALR/CXCL12/NFATC1/NFATC4/PDGFRA/PTGER3/RAC3/TAP1/TAP2	1.13E-04
Focal adhesion	8/41	IGF1/ILK/PDGFD/PDGFRA/PGF/RAC3/SPP1/THBS1	0.000298
Rap1 signaling pathway	8/41	IGF1/KITLG/NGF/PDGFD/PDGFRA/PGF/RAC3/THBS1	0.000332
Epstein-Barr virus infection	7/41	CALR/CXCL10/IRF9/OAS1/STAT1/TAP1/TAP2	0.001821
Phagosome	6/41	CALR/OLR1/TAP1/TAP2/TFRC/THBS1	0.002541
