# Supplementary material for: Identification of immune-related genes as prognostic factors in bladder cancer
Source: Sci Rep. 2020 Nov 12;10:19695. doi: 10.1038/s41598-020-76688-w (PMC7661532; doi:10.1038/s41598-020-76688-w)
Supplement: Supplementary file 11 — Supplementary Information 11. [file 41598_2020_76688_MOESM11_ESM.pdf]

Supplementary material 11. Primer sequences for RT-PCR

Primer	Sequence 5'-3'
SLIT2 forward	TGGCTATCAGGGAGAAAAGTGTG
SLIT2 reverse	CCGCGATATGGTCTTTGTCAC
MMP9 forward	CCTCT GGAGGTTCGACGTGA
MMP9 reverse	TAGG CTTTCTCTCGGTACTGGAA
STAT1 forward	TGAGGGTCCTCTCATCGTCACT
STAT1 reverse	GGCACAACTGGGTTTCAAAAC
AHNAK forward	GGCTTTACTCTCGGATTCCTCT
AHNAK reverse	GCGCATTAGATCTCAACGTAGT
RAC3 forward	AAGTGAAGAGGGATCGGA
RAC3 reverse	CAGATGACTACCATTTGAGG
RBP7 reverse	TTCCATCCATACCACAAGCACA
RBP7 reverse	AGTGAGTCCAGCCCCTGTTCTT
β-actin forward	GTGATCTCCTTCTGCATCCTGT
β-actin reverse	CCACGAAACTACCTTCAACTCC
