# Supplementary material for: Identification of immune-related genes as prognostic factors in bladder cancer
Source: Sci Rep. 2020 Nov 12;10:19695. doi: 10.1038/s41598-020-76688-w (PMC7661532; doi:10.1038/s41598-020-76688-w)
Supplement: Supplementary file 12 — Supplementary Information 12. [file 41598_2020_76688_MOESM12_ESM.docx]

**Supplementary material 1. Gene functional enrichment analysis of DEGs:** (A and B) GO biological process categories.

**Supplementary material 2. GO biological process categories of differentially expressed genes.**

**Supplementary material 3.The KEGG pathway of DEGs:** (A) The top 10 significantly enriched KEGG pathway. (B) The visual network of the relationship between IRGs and TOP5KEGG pathway.

**Supplementary material 4. KEGG pathway categories of differentially expressed genes.**

**Supplementary material 5. GO biological process categories of differentially expressed immune-related genes (DEIRGs).**

**Supplementary material 6. KEGG pathway categories of differentially expressed immune-related genes (DEIRGs).**

**Supplementary material 7. The relationships between survival-associated IRGs and AJCC stage.** (A) ADIPOQ; (B) AGTR1; (C) AHNAK; (D) ENDRA; (E) RBP7; (F) SLIT2.

**Supplementary material 8. The relationships between survival-associated IRGs and T stage.** (A) ADIPOQ; (B) AGTR1; (C) AHNAK; (D) ENDRA; (E) RBP7; (F) SLIT2.

**Supplementary material 9. The relationships between risk score and gender.** (A) NAMPT; (B) RLN2.

**Supplementary material 10. Transcription factor-mediated regulatory network.**

**Supplementary material 11. Primer sequences for RT-PCR.**
